# EFFECT OF WRIST WRAP IN HANDGRIP STRENGTH IN CROSSFIT

**DOI:** 10.1590/1413-785220233103e266236

**Published:** 2023-09-08

**Authors:** Renan Lyuji Takemura, Carla Calviente Ortolani, Mateus Saito, Ricardo Boso Escudero, João Carlos Nakamoto, Luiz Sorrenti

**Affiliations:** 1 Instituto Vita, Department of Hand and Wrist Surgery, São Paulo, Brazil.

**Keywords:** Sports, Sports Equipment, Wrist, Hand Strength, Esportes, Equipamentos esportivos, Punho, Força da mão

## Abstract

**Objectives:**

Analyze wrist wrap influence on the values of maximum handgrip strength and dynamic resistance.

**Methods:**

A controlled randomized cross-over trial including 54 Crossfit participants randomly assigned to two groups. Group 1 began the series of tests with control wrapping, and Group 2 started with functional wrapping. Alternate series of four dynamic grip strength resistance tests were performed, and the resistance and fatigue values were calculated.

**Results:**

The values obtained from the grip tests did not indicate any effect from the wrist wrap for an increase in maximum grip strength (35.7 vs. 35.6 kg; p=0.737) or greater endurance (78.2 vs. 77.8%; p=0.549). Fatigue was also equal in both groups (mean differences between the groups: 0.1 kg, CI= -0.7–0.8; p=0.779).

**Conclusion:**

The hypothesis that using a wrist wrap increases maximum strength and dynamic handgrip endurance was rejected in this study. ***Evidence Level I; Randomized control trial*** .

## RESUMO

**Objetivos:**

Analisar a influência do uso da munhequeira no valor máximo de força e na resistência dinâmica de preensão palmar.

**Métodos:**

Foi feito um ensaio controlado randomizado cruzado (cross-over) incluindo 54 praticantes de Crossfit. Os participantes foram alocados em dois grupos de forma aleatorizada. O grupo 1 iniciou a bateria de testes com enfaixamento placebo e o grupo 2 iniciou com enfaixamento funcional. Séries alternadas de quatro testes dinâmicos de resistência foram realizadas e os valores de resistência e fadiga foram calculados.

**Resultados:**

Os resultados apontaram para uma ausência de efeito do enfaixamento do punho tanto para um suposto aumento da força máxima de preensão (35,7 vs 35,6 kg; p=0,737) quanto para uma maior resistência (78,2 vs 77,8%; p=0,549). A fadiga também foi igual entre os dois grupos (média das diferenças entre os grupos: 0,1kg, CI: -0,7 – 0,8; p=0,779).

**Conclusão:**

A hipótese de que o uso da munhequeira aumenta o valor máximo de força e a resistência dinâmica de preensão palmar foram rejeitadas neste estudo. ***Nível de evidência I; Estudo clínico randomizado*** .

## INTRODUCTION

Crossfit is a training and conditioning program that has gained significant recognition and popularity worldwide among the physically active population. It is based on a set of exercises, including running, Olympic weightlifting, Olympic gymnastics and ballistic movement.^1^ In this context, having greater manual grip strength would allow lifting more weight, and greater grip resistance would secure the weight longer and increase the number of repetitions of certain movements, improving performance.

Many Crossfit participants use wrist wrap during training because it is believed to increase grip strength. However, despite being widely used by Crossfit participants, little is known about the effects of using wrist wraps on the hand regarding grip strength. The idea that using a wrist wrap can increase grip strength is not new.^2^ In 1997 Rettig et al. in a study with young American football athletes, showed that wrapping the wrist did not increase maximum hand grip strength and, considering only the dominant side, even decreased in value^(2)^. Two more recent studies, in 2014 and 2013,^3 , 4^ showed that pressure exerted on the wrist may not influence the maximum grip strength value and may even reduce it, depending on the pressure and the properties of the material used to compress the wrist. As for dynamic grip strength resistance, the most important aspect and one that would directly influence performance, we did not find any data in the literature that measured the influence of the wrist wrap.

The objective of this test is to hypothesize that using a wrist wrap can increase the maximum grip strength and endurance.

## METHODS

### Study design

A controlled randomized cross-over trial was evaluated and approved by the Institutional Review Board of IGESP Hospital (approval 35643920.3.0000.5450). All participants signed the informed consent form. The mean values and standard deviation previously published^5^ were used. The sample size of 54 participants was calculated for this cross-over study to detect a 5kg hand grip difference between the groups in two-sided tests with 80% power and a 5% significance level.

### Inclusion and exclusion

Participants were screened in a CrossFit training center. The inclusion criteria were: to be an amateur Crossfit athlete with at least six months of regular sports practice (at least four times a week), between 18 and 40 years, agree to participate in the study, and sign the informed consent form. The exclusion criteria were: active pain on the day of the test (VAS scale pain score higher than 3 out of 10 in the shoulder, forearm, wrist and/or hand), history of wrist injury in the previous six months, previous wrist surgery.

### Study groups

**Figure 1:**
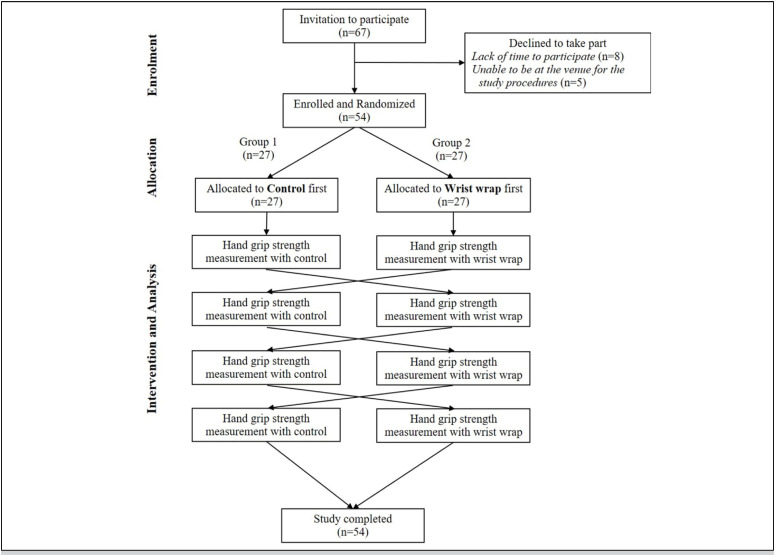
Flow chart diagram of the current study.

Because this is a cross-over study, all participants underwent tests with the control and functional wrapping (intervention; Figure 1 ). To minimize the confounding effect of the order that would be defined at the beginning of the tests, the participants were randomly separated into two study groups (1:1 ratio): Group 1, performing the test with the control wrapping first, and Group 2, completing the functional wrapping test (intervention) first. The random sequence was generated using the GraphPad online program (GraphPad Software(R), San Diego, USA). A single author was responsible for generating the random allocation sequence and only revealed the allocation of a participant when the other author successfully completed an enrollment. Due to the required procedures, it was not possible to blind neither the researcher nor the participant.

### Procedures and data collection

**Figure 2:**
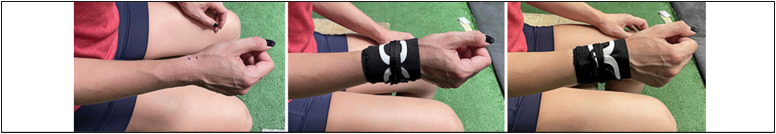
Wrist warp. On the left, two wrist markings show the radial styloid and the 1 cm distal point; in the center, it is shown the application of the control wrist wrap; and on the right, the application of the functional wrist wrap.

Tests were performed in a CrossFit training center. Fabric wrist wraps 35 inches in length by 3 inches in width (Rogue Fitness®, Columbus, USA) were used as test intervention in both groups in this study. As no user manual is available, the researcher applied them standardized to simulate the conditions under which they are normally used in sports. A mark was made 1 cm distal to the radial styloid, and the wrist wrap was positioned, so the distal edge was aligned with the mark ( Figure 2 ). The wrapping pressure was applied up to a tight enough level but without causing discomfort to the participant. The same wrapping procedure was performed for the control application, but no pressure was applied asrecommended above.

The tests were conducted 1 hour before the participant's usual training time using the dominant wrist. The participant was given a standardized orientation about the test and performed a warm-up that included wrist mobility and moderate grip strength exercises.

**Figure 3:**
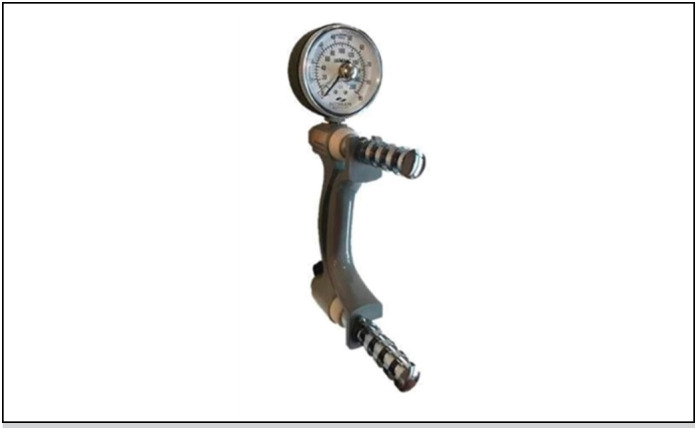
Manual pressure dynamometer used in the tests.

**Figure 4:**
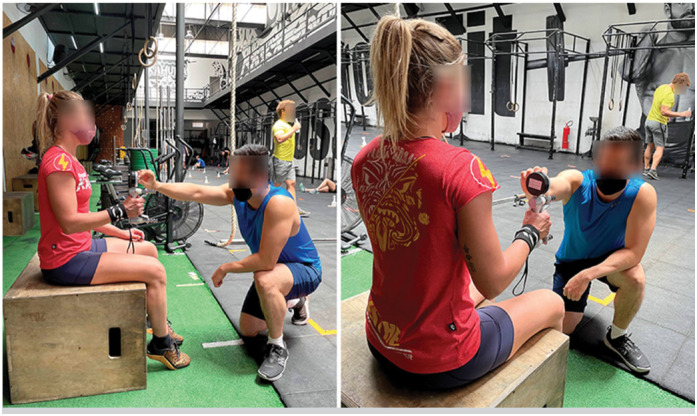
Positioning of the participant and the examiner during the test.

We followed the recommendation of the American Society of Hand Therapists for the grip test,^6^ using a portable dynamometer (Jamar, 5030J1; Jamar Technologies, Horsham, PA) ( Figure 3 ) calibrated before the study. The participant was seated with shoulders abducted and in neutral rotation, the elbows flexed at 90 degrees, the forearms in a neutral position, and the wrist extended between 0 and 30 degrees ( Figure 4 ). The dynamometer grip position was adjusted to each participant's hand size, and this position was always maintained. Once the participant could distinguish between a loose and tight wrap, it was impossible to blind the volunteers regarding the study group.

The examination was conducted in 4 consecutive tests with a 5-minute wash-out interval spaced using the control and functional wrappings, randomly determining whether the participant would perform the first test under control or intervention conditions (Group 1 or Group 2). Each test consisted of 12 contractions of 3 seconds with 5 seconds of rest between repetitions following the 2016 study by Gerodimos et al.^7^ . After each dynamometer reading, the examiner shared the results verbally with the participant for their feedback.

The maximum strength data (kilograms) of the first three and last three contractions in each test were collected. The maximum grip value was the mean of the maximums attained in the three initial movements. The endurance value is the percentage of grip maintenance achieved in the last three movements (fatigue = 100% – endurance). The mean values of the two intervention and the two control tests of each participant were used for data analysis.

### Statistical analysis

Statistical analysis was performed using descriptive methods and comparing the control and wrist wrap data. Normality tests were conducted to infer the distribution of the parameters obtained. As a cross-over trial, each participant was the control subject for themselves for all hypothesis testing, so no normalization for body mass index or other parameters was necessary. The Wilcoxon test for paired samples and the Mann-Whitney U test for independent samples was used. Fisher's exact test was used to analyze differences for categorical variables. The data were compiled in Excel tables (Office 16, Microsoft) and analyzed statistically using the SPSS 16.0 software (IBM SPSS).

## RESULTS

Fifty-four Crossfit participants were successfully included between September 2020 and February 2021: mean age of 32.9 years old (4.8, standard deviation, SD) and 48% female. Athletes were randomized, resulting in groups with similar baseline parameters. ( Table 1 )

**Table 1:** Demographic data and group comparison.

	Group 1	Group 2	p value
Volunteers (“n”)	27	27	-
Sex (F/ M)	14/13	12/15	0.786
Age (mean ± SD)	32.9 ± 5.0	33.4 ± 4.6	0.664
Right hand dominance	25	24	0.999

F: female; M: male; SD: standard deviation

No volunteers were lost during the study, and all of them completed the series of 4 tests with both wrapping configurations (control and functional wrist wrap; Figure 1 ).

The results from the tests pointed to the absence of any effect resulting from wrist wrapping ( Table 2 ), either for a supposed increase in initial strength (grip) or for greater resistance/decreased fatigue.

**Table 2:** Results from test with hand grip.

	Control (n=54)	Wrist Wrap (n=54)	Effect size	p value
	mean ± SD	mean ± SD	mean (95%CI)	
Grip (kg)	35.7 ± 8.6	35.6 ± 8.3	-0.1 (-0.7–0.6)	0.737
Endurance (%)	78.2 ± 6.7	77.8 ± 7.4	0.0 (-0,01–0.02)	0.549

SD: standard deviation; CI: confidence interval. Effect size shown as mean differences

## DISCUSSION

In this study, using a wrist wrap did not interfere with maximum strength or grip resistance. The lack of an increase in maximum grip strength is in line with other studies that tested athletes^8^ and others that specifically studied wrist use. In a cross-over clinical trial,^9^ Johansson et al. demonstrated that maximum grip strength did not vary with a commercial wrist band, similar to Rettig et al.,^2^ who used adhesive tape. Although Takahashi et al. demonstrated the possibility of altering hand grip strength by compressing the wrist,^4^ this outcome only occurred above a certain pressure level.

About muscle fatigue, contractions to measure grip cause blood flow to be intermittent, staying impeded by the pressure during the contraction. According to Pitcher and Mies,^10^ this restriction of the blood would contribute to muscle fatigue in the forearm. However, the author reported changes only after two minutes of vascular occlusion, which is longer than the total intervention time of each test. In addition, in our intervention, the compression was generated only in the wrist, where there is less muscle mass, unlike this author, who used an arm cuff.

Another reason the intervention increases fatigue is the greater effort of the wrist extensor muscles, given the limitation of dorsiflexion caused by the wrist wrap. Di Domizio et al. demonstrated that a wrist orthotic increases activation of the extensor muscles when 100% grip strength is required.^11^ This fatigue also can interfere with grip strength^(12)^, though in our test, there was no increase in fatigue.

Certainly, one of the strong points of our study is its cross-over design and randomized allocation for the first trials. Among the confounding factors are the muscle fatigue that interferes progressively in consecutive tests, decreasing the maximum grip strength values, and the learning during the series of tests, since the candidate tends to optimize their strength and therefore have better results in the consecutive tests after being better familiarized with the effort required for the task. In addition, we had any loss of participants for the retest.

We also highlight the pioneering nature of this study, as there is little scientific research involving Crossfit participants. To date, there are only a few clinical trials involving Crossfit.

Not measuring the pressure exerted on the wrist during intervention is a limitation of this study. As previously mentioned, Takahashi and Demura demonstrated that pressure applied to the wrist could interfere with maximum grip strength when above 90hPa.^3^ Another limitation of the study is that it did not control pressure on the wrist when submitted to intervention. As pressure adjustment was subjective, allowing the participant to self-adjust according to their comfort level could have caused significant variation among each patient's tests. Also, some participants’ unfamiliarity with using the wrist wrap could have increased this variability.

Even though the study demonstrated that the wrist wrap did not impact the maximum grip strength or the dynamic grip resistance, it is not possible to state that it does not affect performance since there are different effects of the wrist wrap that could impact performance. Kauranen et al. proved that wrapping the wrist improves the participant's agility by reducing simple reaction time and choice reaction time in a standardized performance test.^13^

Another possible effect of using wrapping is improved proprioception. Karagiannopoulos et al. demonstrated that the sense of wrist joint positioning, which can deteriorate naturally after exercise-induced muscle fatigue,^14^ can be improved with adhesive tape,^15^ an intervention similar to our wrist wrap in terms of its positioning on the wrist.

Kim et al. demonstrated how wrapping the wrist could increase the range of motion of wrist extension associated with axial load in individuals with a reduced arc of wrist extension motion.^16^ Considering that many Crossfit exercises associate movements of maximum wrist extension with axial load (for example handstand walking, snatch, clean and jerk, etc.), it is possible that the wrist wrap has a similar effect and allows a greater arc of motion in people with reduced wrist mobility, which would have a direct impact on the performance of the participant.

## CONCLUSION

We concluded in our study that using a wrist wrap does not affect the maximum hand grip strength and resistance. However, the effect of the wrist wrap on the Crossfit performance was not studied in a more global context, and it may be a topic of investigation in future studies.
